# Rural residents’ Knowledge, Attitude, and Practice in relation to infection risk during the late stage of an epidemic: a cross-sectional study of COVID-19

**DOI:** 10.3389/fpubh.2024.1450744

**Published:** 2024-12-04

**Authors:** Manting Zhuang, Lixiang Zhai, Hui Zhang, Qingsong Chen, Ran Xiong, Yonghui Liu, Fangyi Zhu

**Affiliations:** ^1^School of Public Health, Guangdong Pharmaceutical University, Guangzhou, China; ^2^Guangdong Health Economics and Health Promotion Research Center, Guangzhou, China

**Keywords:** Knowledge, Attitudes, Practices, COVID-19, SARS-CoV-2 infection, rural residents

## Abstract

**Background:**

In the field of public health, the prevention and management of infectious diseases in rural regions have always been crucial. This study aims to analyze the factors influencing rural residents’ Knowledge, Attitude, and Practices and their correlation with infection risk during the late stage of an epidemic, with a focus on the COVID-19 case.

**Methods:**

A cross-sectional study was conducted in rural regions of China’s Guangdong province, using a multi-stage sampling technique to select rural residents for a validated questionnaire survey in February 2023. Descriptive statistical method was used to describe the infection status of rural residents and Chi-Square Test was used to explore the influencing factors of Knowledge, Attitude and Practice in this population. Multivariable binary logistic regression analysis was conducted to determine the presence of a statistically significant association between explanatory variables and outcome variables at corresponding 95% CI.

**Results:**

A total of 3,125 rural residents were investigated, of whom 805 had never been infected with COVID-19. The survey participants had an average score of 5.84 ± 1.419 for COVID-19 knowledge. (The total score range is from 0 to 8. A score greater than 6.4 indicates good knowledge acquisition.) Regarding the attitude and practice sections, the average scores were 23.68 ± 3.169 and 23.45 ± 5.030, respectively. (The total score range of both these sections is from 0 to 32. A score greater than 25.6 represents positive attitudes and good practices.) The reduction of COVID-19 risk is significantly associated with an increase in Knowledge scores (*p* trend < 0.01). In stratified analyses, the Knowledge, Attitudes, and Practices scores of residents in each region have varying degrees of correlation with the risk of SARS-CoV-2 infection.

**Conclusion:**

Rural residents’ Knowledge, Attitudes, and Practices on COVID-19 prevention and control requires improvement. Efforts to promote their’ perceptions and habits regarding COVID-19 prevention and control are crucial in reducing the risk of infection.

## 1 Introduction

A string of severe infectious disease outbreaks has been reported in the twenty-first century, most notably the global devastation caused by the COVID-19 pandemic (1). SARS-CoV-2 is a newly discovered pathogenic virus which can cause serious respiratory diseases, namely novel coronavirus (2, 3). Rapid country-to-country transmission of the COVID-19 outbreak has elevated it to a public health emergency of global concern (4–6). The SARS-CoV-2 infection in rural locations has brought about unique challenges and traits (7–9). The aftermath of the COVID-19 pandemic underscores the ongoing need for a comprehensive understanding of how Knowledge, Attitude, and Practices (KAP) among rural residents correlate with infection risk, even as the initial crisis de-escalates. While extensive research has been conducted on COVID-19 (10–14), there remains a knowledge gap regarding the correlation between infection rates and the KAP of prevention and control among rural residents in the later stages of the epidemic in China.

In December 2019, COVID-19 was first identified in Wuhan, Hubei. Within 2 weeks, the disease spread rapidly from Hubei province to other provinces in China. Since January 13, 2020, over 200 countries have reported imported cases of COVID-19. On March 12, 2020, the WHO formally declared that COVID-19 had entered the worldwide epidemic phase and classified it as a pandemic (15). Since December 9, 2022, the number of positive cases and the positivity rate of SARS-CoV-2 nucleic acid tests reported across China’s provinces have exhibited a trend of initial increase followed by a decline. The peak number of positive cases was reached on December 22, amounting to approximately 6.94 million, which subsequently experienced a fluctuating decrease to 3,575 cases by March 23, 2023. In rural areas, the impact of the epidemic also showed a similar trend. On December 23, 2022, the number of people attending fever clinics in township hospitals nationwide reached a peak of 922,000, and by February 23, 2023, this number had dropped to 33,000, a decrease of 96.4% from the peak. Within this context, the situation in Guangdong Province mirrored the national trend. In response to the pandemic, the National Health Commission of China, through the Joint Prevention and Control Mechanism of the State Council, has underscored the critical role of vaccination as a fundamental measure for the prevention and control of SARS-CoV-2 infections. Guangdong Provincial Government issued the “Notification on the Issuance of the Work Plan for COVID-19 Vaccination, “targeting the reduction of immunization gaps among different population groups to further mitigate the risks of severe disease and mortality. These measures have been instrumental in curbing the spread of the virus and facilitating the gradual restoration of social order and economic activities across the nation, including in Guangdong Province.

Unlike metropolitan regions, rural populations possess distinct characteristics, such as limited access to healthcare services (16), lower health literacy (17, 18), and cultural variations that impact disease prevention and control (19). The spread of COVID-19 epidemic in rural regions highlights the challenges faced by rural regions, including scarce medical resources, population migration and insufficient preventive measures (20). Factors like geographic location, uneven population distribution, limited economic development and restricted access to information contribute to the need for additional public health resources, efficient medical service, and increased awareness of self-protection techniques in rural regions (21, 22). During the COVID-19 pandemic, these challenges have been particularly severe, as evidenced by data from the National COVID Cohort Collaborative (N3C), which indicates that rural communities have experienced higher rates of SARS-CoV-2 infection and worse health outcomes, such as hospitalization and mortality rates, compared to urban areas. The disparities in COVID-19 mortality rates between rural and urban areas further underscore the inadequacies in health policy and preparedness in rural regions (23). Given the unique characteristics of rural areas and the significant impact of infectious disease outbreaks on these communities, there is a heightened need to focus on the vulnerabilities of rural populations in the later stages of an epidemic. As previous studies have shown, KAP play a crucial role in public health (24, 25). A better understanding of COVID-19 helps rural populations recognize risks and take appropriate preventive measures to prevent it from evolving into a pandemic. Given the uniqueness of rural areas and the significant impact of infectious disease outbreaks on these communities, there is an urgent need to focus on the vulnerabilities of rural populations in the later stages of an epidemic.

The survey in rural Guangdong, China, provides an important basis for understanding the relationship between KAP and risk of infection after an outbreak, and helps to develop targeted public health strategies for future outbreaks of similar nature in rural China. Guangdong Province is one of the most populous provinces in China and has a diverse range of rural regions representing different socioeconomic conditions and infrastructure levels. By investigating the infection situation in Guangdong’s rural regions, researchers can gain insights into the challenges and patterns that may be applicable to other rural regions across China. In addition, Guangdong has a history of infectious illness outbreaks, including the SARS epidemic in 2003 (26). Since then, the province has established a strong public health system and response framework, which may be used as a valuable case study to learn how rural resident has prepared for and responded to infectious diseases. Overall, in terms of representing various socioeconomic conditions and learning from previous experiences, using rural Guangdong Province as an example to comprehend the infection and response of infectious diseases among rural residents offers significant advantages.

Understanding and tackling the spread of infectious illnesses can benefit from analyzing the relationship between infectious disease infection and KAP scores (27). On the one hand, KAP scores reflect the level of knowledge, attitudes, and practices of people living in rural regions in terms of disease prevention and control. On the other hand, knowing how infection rates and KAP scores are related can assist in pinpointing particular regions or communities that are more susceptible to infectious diseases. This information may guide how resources are allocated, for example, by concentrating healthcare and educational initiatives in fields where knowledge and procedures are inadequate or ineffective. The efficiency of current prevention and control strategies may also be determined by looking at the relationship between infection and KAP scores.

Assessing KAP relative to infection risk in the late stage of an epidemic is crucial for validating the effectiveness of public health interventions and for guiding future preventative measures. Taking COVID-19 as an example, this study aims to analyze the factors affecting rural residents’ Knowledge, Attitude and Practice in the later period of the epidemic and their correlation with infection risk, so as to provide important new perspectives for rural resource allocation, public health policies and public health methods.

## 2 Materials and methods

### 2.1 Study design and participants

This cross-sectional study started on February 1st, 2023 and ended on February 28th, 2023. The survey was conducted in the form of filling in an electronic questionnaire or a paper questionnaire on the spot. And the questionnaire was administered by trained and qualified investigators who conducted face-to-face interviews with survey respondents. The study was conducted under the guidance and assistance of experts from the School of Public Health and the School of Pharmaceutical Business, Guangdong Pharmaceutical University. During the assessment and approval process, they conducted a rational review to ensure that ethical principles were fully considered. In accordance with the ethical guidelines in the Declaration of Helsinki, all participants provided written informed consent before participating in the study. Participants’ anonymity and confidentiality were ensured. On this basis, the ethical risks of the study were minimal and approval was obtained from the School of Pharmaceutical Business, Guangdong Pharmaceutical University and no further ethical review was required. Data entry and statistical analysis were carried out after the questionnaire was recovered and audited.

**Figure 1:**
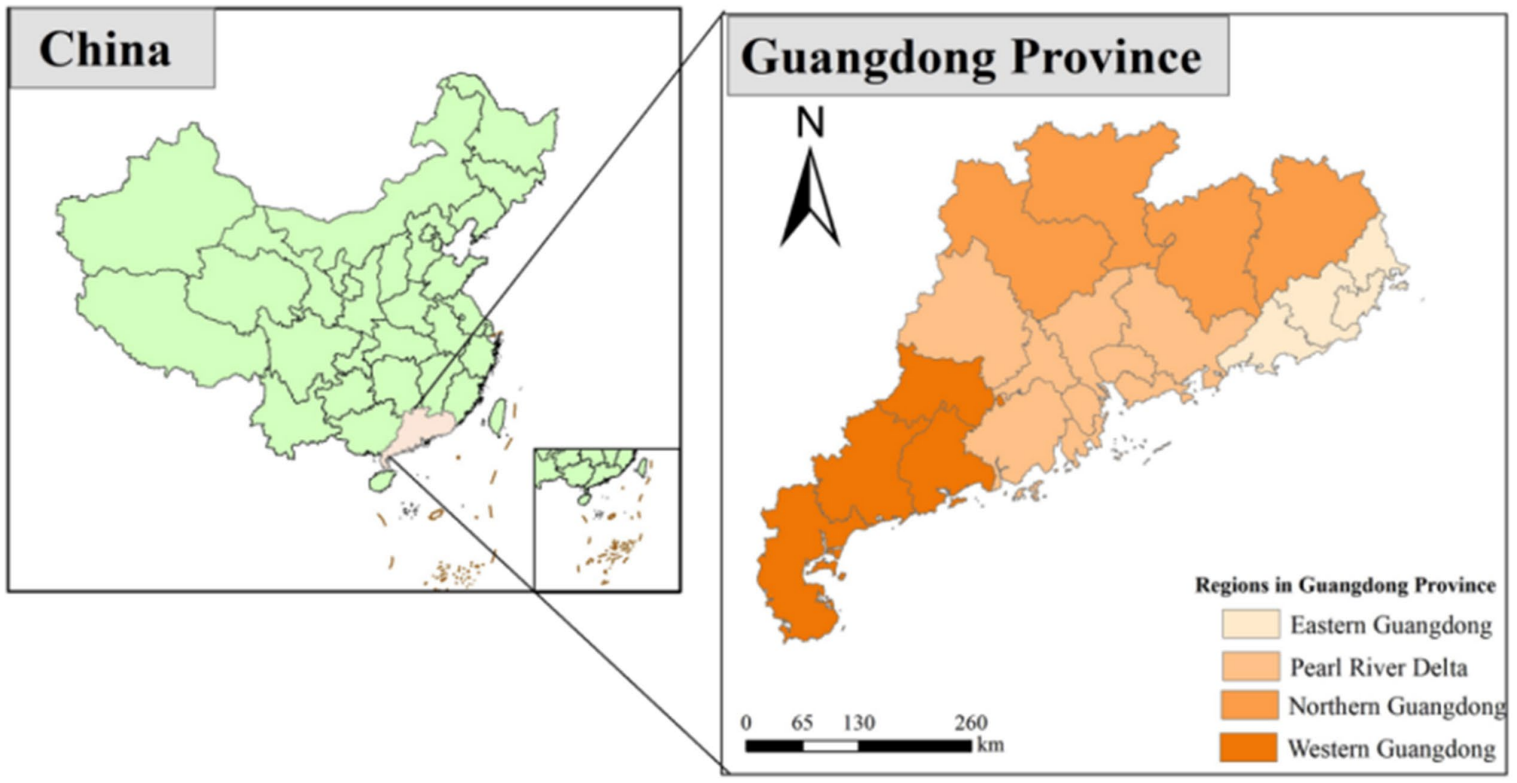
Geographical map of Guangdong Province, China.

A multi-stage sampling approach was adopted in this survey, targeting residents from 23 villages located in the rural regions of Guangdong province in China (Figure 1). A single population proportion formula (*n* = *Z*^2^ *p*(1 − *p*)/*d*^2^) was utilized to compute the sample size. Given the absence of published data indicating the Knowledge, Attitude, and Practice regarding COVID-19 among rural residents in Guangdong Province, a prevalence of 50% was employed to obtain the maximum sample size by taking into account a 95% confidence interval, a marginal error (*d*) of 3%, and a 15% non-response rate. Consequently, the minimum calculated sample size was 2,511. During the implementation process, the sample size of each region was adaptively adjusted according to the actual situation to ensure data quality and the feasibility of the study. The specific sampling process is as follows: In the first stage, based on the proportion of permanent residents in various regions of Guangdong Province in 2021 (the Pearl River Delta region accounts for 61.97%, the eastern Guangdong region accounts for 13.43%, the western Guangdong region accounts for 14.55%, and the northern Guangdong region accounts for 10.05%), probability proportional to size sampling (PPS) was used to determine the sample size of each region. In the second stage, 2–4 cities were randomly selected in each region. In the third stage, according to the established sample size and village scale, several villages were selected from the selected cities. In the selected villages, residents who meet the inclusion criteria are included in the survey scope. Finally, 3,125 valid paper and electronic questionnaires were gathered in total. The inclusion requirements were: (1) living in rural Guangdong Province for at least 6 months; (2) communication is barrier-free; (3) obtaining the respondents’ informed agreement prior to the investigation. Exclusion criteria: Having severe organic illnesses, mental illnesses, consciousness abnormalities, or other illnesses that made it difficult for them to answer questions.

### 2.2 The questionnaire

This research was grounded in the Theory of Knowledge, Attitudes, and Practices (28). Insights from previous relevant studies were incorporated, and expert guidance was sought to independently develop the survey questionnaire. Four lecturers aided the research team in reviewing this questionnaire and checking the plainness and clarity of each question. According to the SPSS reliability test, the questionnaire had strong internal consistency (the Cronbach’s coefficients for the attitude and practice dimensions are 0.721 and 0.841, respectively). A pilot study was conducted with the participation of 30 residents to check the logic and suitability of the questionnaire (the pre-testing of the questionnaire).

The first section of the final questionnaire comprised a brief introduction to the investigation, the declaration of anonymity and confidentiality from researchers, and residents’ confirmation of voluntary participation. The second section included 13 questions regarding the residents’ personal information (such as sex, age, and health status) as well as infection information. Specifically, the infection risk was treated as a binary outcome. Participants were asked if they had tested positive for COVID-19, to which they could respond “Yes” or “No.” The final section consisted of KAP questions, with 8 questions in each section. The questionnaire consists of two types of items: Knowledge section are formatted as single-choice, while Attitude and Practice section are designed as Likert-scale items. There was only one correct response to each question in the Knowledge section, and the correct response was scored, giving the question a total score of 8 points. The 4-point Likert scale was used to calculate scores for the Attitude and Practice section, both of which totaled 32 points. Higher scores on the index reflected better knowledge and indicated more positive attitudes and practices toward the subjects. Using Bloom’s cut-off point, we considered a high level of knowledge, positive attitudes, and good practices to be present when components of the KAP scored no less than 80% of the total score (29, 30). Specifically, for Knowledge section, a score between 80 and 100% (6.4–8 points) is considered a high level of awareness. For Attitudes and Practices sections, a score between 80 and 100% (25.6–32 points) is considered positive attitudes and good practices.

### 2.3 Statistical analysis

The accuracy and completeness of the data were checked once all of the participants had finished the questionnaire. Before data analysis, data were cleaned and checked, and questionnaires with obvious logical errors were excluded. EpiData 3.1 software was used to input data, which was collated and stored as an Excel file after verification. After filtering out invalid or incorrect data, SPSS 26.0 software was used to analyze the data. Participants’ demographics and research variables were described using descriptive statistics. Frequencies and percentages for categorical variables were calculated. Chi-Square Test was used to determine the relation between infection rate toward COVID-19 and socio-demographic variables. Multivariate binary logistic regression analysis was performed to identify statistically associated variables with outcome variables. When analyzing the correlation between KAP and infection risk, KAP scores were divided into four groups according to quartile interval. Taking the Q1 group as a reference, binary logistic regression analysis was used to analyze the correlation between KAP scores and SARS-CoV-2 infection in the original model and two models after adjusting social demographic information (Model 2 and Model 3). Stratified analyses were used to evaluate correlation between KAP score and infection risk in different regions. Variables with *p* values less than 0.05 were considered statistically significant.

## 3 Results

### 3.1 Sociodemographic characteristics of the respondent

The socio-demographic characteristics of the respondents are presented in Table 1. A total of 3,125 people participated in the survey, of which 56.3% were women, 43.7% were men, and 805 (25.8%) were never infected. The mean age (±SD) of the respondents was 34 (±19.8) years. The survey region was divided into four zones based on geographical geography, with the Pearl River Delta accounting for more than half of the respondents (52.2%). Most of the respondents had more than three family members, and the annual *per capita* household incomes were mostly 10,001–15,000 yuan (25.4%) and >20,000 yuan (23.9%). More than four-fifths of people had never smoked (80.4%), and 78.0% had never drunk. There were 403 respondents with chronic illnesses, accounting for 12.9% of the total, and almost 70% of persons were in excellent (33.2%) or good (41.3%) health. Approximately 22.7% of respondents hold a primary school degree or below, while roughly a quarter of them had attained a university education or higher (24.9%). 66.8% people were vaccinated with three doses of COVID-19 vaccine, while 1.6% had received four doses. Over 50% of the participants had proactively prepared infection medications ahead of time (52.1%).

**Table 1:** Baseline characteristics of all participants.

Characteristic		Total sample (*N* = 3,125)	Non-infected group (*N* = 805)	Infected group (*N* = 2,320)	*p*
Region	Pearl River Delta	1,632 (52.2%)	437 (54.3%)	1,195 (51.5%)	0.005
	Eastern Guangdong	502 (16.1%)	149 (18.5%)	353 (15.2%)	
	Western Guangdong	571 (18.3%)	120 (14.9%)	451 (19.4%)	
	Northern Guangdong	420 (13.4%)	99 (12.3%)	321 (13.8%)	
Sex	Male	1,366 (43.7%)	407 (50.6%)	959 (41.3%)	<0.001
	Female	1,759 (56.3%)	398 (49.4%)	1,361 (58.7%)	
Age (years)	<19	780 (25.0%)	218 (27.1%)	562 (24.2%)	<0.001
	19~	744 (23.8%)	204 (25.3%)	540 (23.3%)	
	27~	806 (25.8%)	159 (19.8%)	647 (27.9%)	
	49~	795 (25.4%)	224 (27.8%)	571 (24.6%)	
Number of household members	<4	752 (24.1%)	217 (27.0%)	535 (23.1%)	<0.001
	4~	914 (29.2%)	187 (23.2%)	727 (31.3%)	
	5~	1,459 (46.7%)	401 (49.8%)	1,058 (45.6%)	
Annual *per capita* household incomes (RMB)	≤5,000	598 (19.1%)	176 (21.9%)	422 (18.2%)	<0.001
	5,001–10,000	545 (17.4%)	141 (17.5%)	404 (17.4%)	
	10,001–15,000	793 (25.4%)	230 (28.6%)	563 (24.3%)	
	15,001–20,000	441 (14.1%)	117 (14.5%)	324 (14.0%)	
	>20,000	748 (23.9%)	141 (17.5%)	607 (26.2%)	
Smoking status^a^	Never	2,513 (80.4%)	627 (77.9%)	1,886 (80.4%)	0.103
	Current	500 (16.0%)	147 (18.3%)	353 (15.2%)	
	Previous	112 (3.6%)	31 (3.9%)	81 (3.5%)	
Alcohol consumption^a^	Never	2,437 (78.0%)	610 (75.8%)	1,827 (78.8%)	0.120
	Current	567 (18.1%)	156 (19.4%)	411 (17.7%)	
	Previous	121 (3.9%)	39 (4.8%)	82 (3.5%)	
Chronic diseases	Yes	403 (12.9%)	108 (13.4%)	295 (12.7%)	0.609
	No	2,722 (87.1%)	697 (86.6%)	2,025 (87.3%)	
Health status	Excellent	1,037 (33.2%)	301 (37.4%)	736 (31.7%)	0.001
	Good	1,287 (41.2%)	332 (41.2%)	955 (41.2%)	
	Fair	694 (22.2%)	157 (19.5%)	537 (23.1%)	
	Poor	91 (2.9%)	11 (1.4%)	80 (3.4%)	
	Very poor	16 (0.5%)	4 (0.5%)	12 (0.5%)	
Education level	Primary school and below	708 (22.7%)	201 (25.0%)	507 (21.9%)	0.128
	Junior high school	1,024 (32.8%)	268 (33.3%)	756 (32.6%)	
	High school/technical secondary school	616 (19.7%)	157 (19.5%)	459 (19.8%)	
	University/junior college and above	777 (24.9%)	179 (22.2%)	598 (25.8%)	
Vaccination in COVID-19	One dose	53 (1.7%)	20 (2.5%)	33 (1.4%)	<0.001
	Two doses	825 (26.4%)	218 (27.1%)	607 (26.2%)	
	Three doses	2,087 (66.8%)	505 (62.7%)	1,582 (68.2%)	
	Four doses	50 (1.6%)	21 (2.6%)	29 (1.3%)	
	Not vaccinated	110 (3.5%)	41 (5.1%)	69 (3.0%)	
Preparation of drugs in advance	Yes	1,628 (52.1%)	387 (48.1%)	1,241 (53.5%)	0.008
	No	1,497 (47.9%)	418 (51.9%)	1,079 (46.5%)	

a Confined to the past 3 months.

The demographic characteristics of individuals with different infection status were analyzed by Chi-Square Test. Infection rate of COVID-19 was significantly associated (*p* < 0.001) with sex, age, family population, annual *per capita* household incomes, dose of COVID-19 vaccine, health status (*p* = 0.001), preparation of drugs in advance (*p* = 0.008), region (*p* = 0.005), but not smoking status (*p* = 0.103), alcohol consumption (*p* = 0.120), chronic diseases (*p* = 0.609), and education level (*p* = 0.128).

### 3.2 Knowledge, Attitude and Practice toward COVID-19

#### 3.2.1 Knowledge level of COVID-19 and its influencing factors

**Figure 2:**
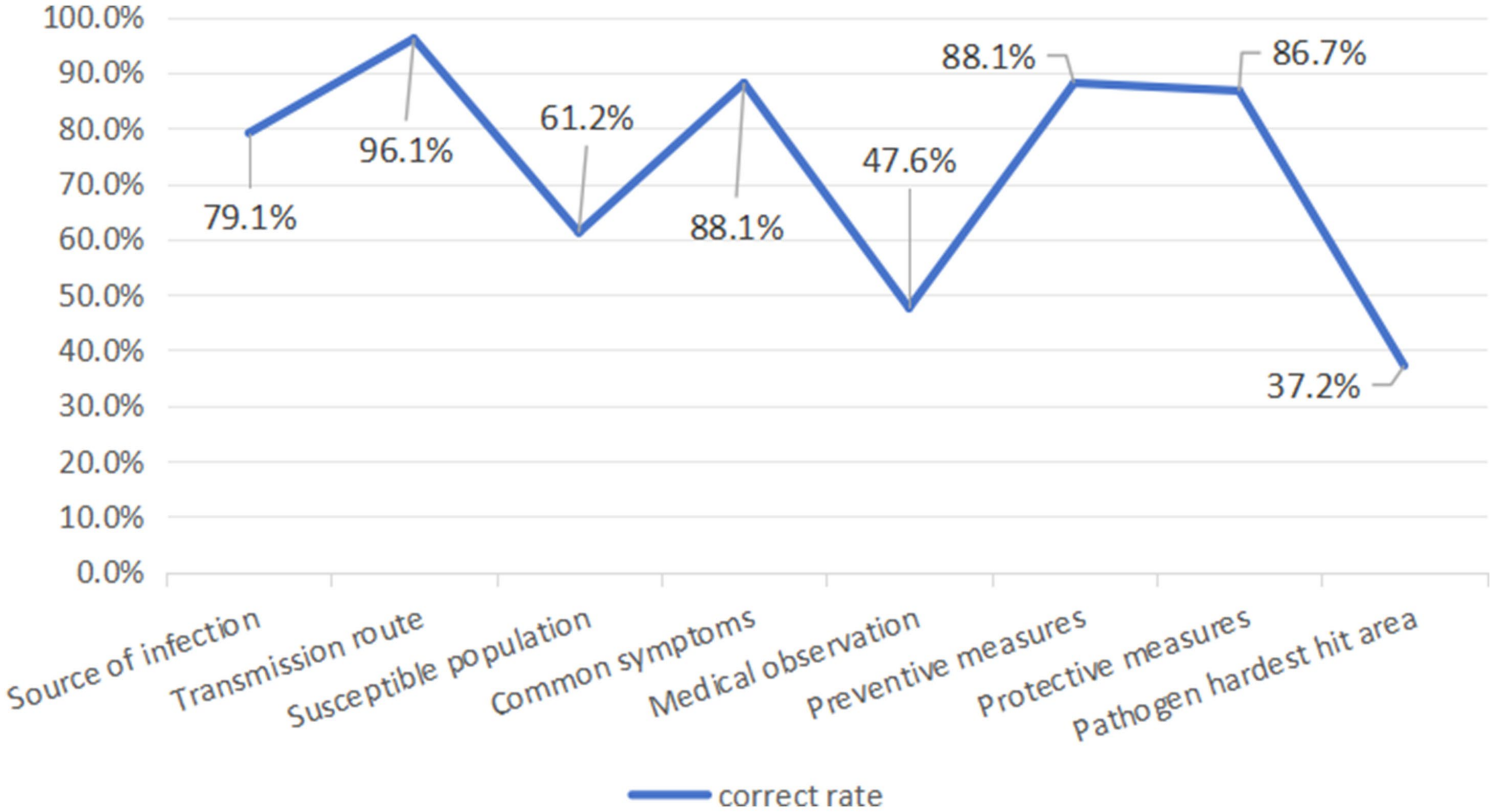
Correct answer rate of COVID-19 knowledge questions.

The total score of Knowledge about COVID-19 was 8, and the average score of the respondents was 5.84 ± 1.419, with 37.7% having high Knowledge level. The majority of respondents accurately identified the symptoms, prevention strategies, and modes of transmission of COVID-19, the disease caused by SARS-CoV-2, while the medical observation duration was the knowledge item with the highest mistake rate. The findings of the Chi-Square Test indicated that there were no statistically significant differences in the respondents’ Knowledge levels with respect to sex, alcohol consumption and smoking status, and chronic conditions (*p* > 0.05). There was a balanced distribution of Knowledge levels between rural residents in northern Guangdong and those with annual *per capita* household incomes between 10,001 and 15,000 yuan, with high Knowledge level accounting for 49.0 and 47.2%, respectively. The disparity in Knowledge levels among respondents aged 49 and above was most apparent as seen by only 33.3% of respondents exhibiting a high degree of knowledge. The Knowledge level of residents with different health conditions was quite different. For those who were in very poor health in the past year, the high level of Knowledge only accounted for 18.8% of the population. Respondents who had completed four doses of the COVID-19 vaccination, attended high school or a technical secondary school, and had prepared their medications in advance were more likely to demonstrate a high level of understanding (Figure 2; Table 2).

**Table 2:** Participants’ KAP score levels and influencing factors.

Characteristic		Knowledge scores level		*p*	Attitude scores level		*p*	Practice scores level		*p*
		Low	High		Low	High		Low	High	
Region	Pearl River Delta	978 (59.9%)	654 (40.1%)	<0.001	1,157 (70.9%)	475 (29.1%)	<0.001	1,180 (72.3%)	452 (27.7%)	<0.001
	Eastern Guangdong	344 (68.5%)	158 (31.5%)		399 (79.5%)	103 (20.5%)		355 (70.7%)	147 (29.3%)	
	Western Guangdong	412 (72.2%)	159 (27.8%)		375 (65.7%)	196 (34.3%)		275 (48.2%)	296 (51.8%)	
	Northern Guangdong	214 (51.0%)	206 (49.0%)		267 (63.6%)	153 (36.4%)		274 (65.2%)	146 (34.8%)	
Sex	Male	838 (61.3%)	528 (38.7%)	0.315	973 (71.2%)	393 (28.8%)	0.335	931 (68.2%)	435 (31.8%)	0.125
	Female	1,110 (63.1%)	649 (36.9%)		1,225 (69.6%)	534 (30.4%)		1,153 (65.5%)	606 (34.5%)	
Age (years)	<19	474 (60.8%)	306 (39.2%)	0.002	491 (62.9%)	289 (37.1%)	<0.001	493 (63.2%)	287 (36.8%)	<0.001
	19~	477 (64.1%)	267 (35.9%)		500 (67.2%)	244 (32.8%)		465 (62.5%)	279 (37.5%)	
	27~	467 (57.9%)	339 (42.1%)		609 (75.6%)	197 (24.4%)		530 (65.8%)	276 (34.2%)	
	49~	530 (66.7%)	265 (33.3%)		598 (75.2%)	197 (24.8%)		596 (75.0%)	199 (25.0%)	
Number of household members	<4	481 (64.0%)	271 (36.0%)	0.044	534 (71.0%)	218 (29.0%)	0.687	490 (65.2%)	262 (34.8%)	0.335
	4~	539 (59.0%)	375 (41.0%)		633 (69.3%)	281 (30.7%)		602 (65.9%)	312 (34.1%)	
	5~	928 (63.6%)	531 (36.4%)		1,031 (70.7%)	428 (29.3%)		992 (68.0%)	467 (32.0%)	
Annual *per capita* household incomes (RMB)	≤5,000	443 (74.1%)	155 (25.9%)	<0.001	458 (76.6%)	140 (23.4%)	<0.001	448 (74.9%)	150 (25.1%)	<0.001
	5,001–10,000	388 (71.2%)	157 (28.8%)		400 (73.4%)	145 (26.6%)		380 (69.7%)	165 (30.3%)	
	10,001–15,000	419 (52.8%)	374 (47.2%)		532 (67.1%)	261 (32.9%)		527 (66.5%)	266 (33.5%)	
	15,001–20,000	249 (56.5%)	192 (43.5%)		295 (66.9%)	146 (33.1%)		256 (58.0%)	185 (42.0%)	
	>20,000	449 (60.0%)	299 (40.0%)		513 (68.6%)	235 (31.4%)		473 (63.2%)	275 (36.8%)	
Smoking status^a^	Never	1,552 (61.8%)	961 (38.2%)	0.076	1,752 (69.7%)	761 (30.3%)	0.074	1,669 (66.4%)	844 (33.6%)	0.549
	Current	332 (66.4%)	168 (33.6%)		357 (71.4%)	143 (28.6%)		343 (68.6%)	157 (31.4%)	
	Previous	64 (57.1%)	48 (42.9%)		89 (79.5%)	23 (20.5%)		72 (64.3%)	40 (35.7%)	
Alcohol consumption^a^	Never	1,529 (62.7%)	908 (37.3%)	0.639	1,688 (69.3%)	749 (30.7%)	0.024	1,613 (66.2%)	824 (33.8%)	0.249
	Current	347 (61.2%)	220 (38.8%)		415 (73.2%)	152 (26.8%)		394 (69.5%)	173 (30.5%)	
	Previous	72 (59.5%)	49 (40.5%)		95 (78.5%)	26 (21.5%)		77 (63.6%)	44 (36.4%)	
Chronic diseases	Yes	244 (60.5%)	159 (39.5%)	0.427	298 (73.9%)	105 (26.1%)	0.089	291 (72.2%)	112 (27.8%)	0.012
	No	1,704 (62.6%)	1,018 (37.4%)		1,900 (69.8%)	822 (30.2%)		1,793 (65.9%)	929 (34.1%)	
Health status	Excellent	665 (64.1%)	372 (35.9)	<0.001	669 (64.5%)	368 (35.5%)	<0.001	649 (62.6%)	388 (37.4%)	<0.001
	Good	725 (56.3%)	562 (43.7%)		908 (70.6%)	379 (29.4%)		853 (66.3%)	434 (33.7%)	
	Fair	481 (69.3%)	213 (30.7%)		534 (76.9%)	160 (23.1%)		503 (72.5%)	191 (27.5%)	
	Poor	64 (70.3%)	27 (29.7%)		73 (80.2%)	18 (19.8%)		67 (73.6%)	24 (26.4%)	
	Very poor	13 (81.3%)	3 (18.8%)		14 (87.5%)	2 (12.5%)		12 (75.0%)	4 (25.0%)	
Education level	Primary school and below	485 (68.5%)	223 (31.5%)	<0.001	524 (74.0%)	184 (26.0%)	0.030	559 (79.0%)	149 (21.0%)	<0.001
	Junior high school	598 (58.4%)	426 (41.6%)		725 (70.8%)	299 (29.2%)		679 (66.3%)	345 (33.7%)	
	High school/technical secondary school	353 (57.3%)	263 (42.7%)		428 (69.5%)	188 (30.5%)		396 (64.3%)	220 (42.1%)	
	University/junior college and above	512 (65.9%)	256 (34.1%)		521 (67.1%)	256 (32.9%)		450 (57.9%)	327 (42.1%)	
Vaccination in COVID-19	One dose	38 (71.7%)	15 (28.3%)	0.002	41 (77.4%)	12 (22.6%)	0.121	41 (77.4%)	12 (22.6%)	0.100
	Two doses	548 (66.4%)	277 (33.6%)		557 (67.5%)	268 (32.5%)		532 (64.5%)	293 (35.5%)	
	Three doses	1,258 (60.3%)	829 (39.7%)		1,483 (71.1%)	604 (28.9%)		1,408 (67.5%)	679 (32.5%)	
	Four doses	26 (52.0%)	24 (48.0%)		33 (66.0%)	17 (34.0%)		28 (56.0%)	22 (44.0%)	
	Not vaccinated	78 (70.9%)	32 (29.1%)		84 (76.4%)	26 (23.6%)		75 (68.2%)	35 (31.8%)	
Preparation of drugs in advance	Yes	964 (59.2%)	664 (40.8%)	<0.001	1,128 (69.3%)	500 (30.7%)	0.181	1,034 (63.5%)	594 (36.5%)	<0.001
	No	984 (65.7%)	513 (34.3%)		1,070 (71.5%)	427 (28.5%)		1,050 (70.1%)	447 (29.9%)	

a Confined to the past 3 months.

#### 3.2.2 Attitude level of COVID-19 and its influencing factors

**Figure 3:**
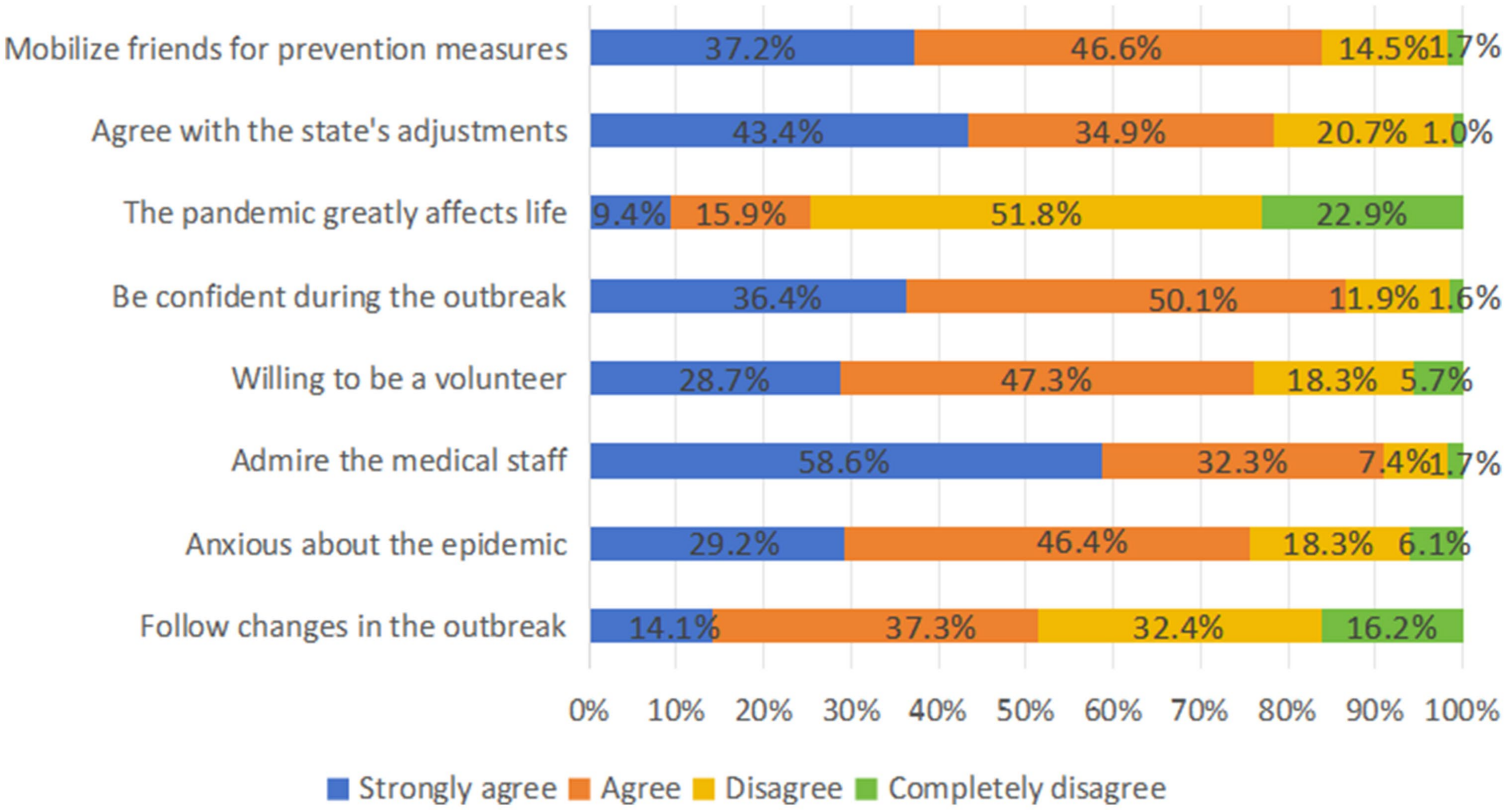
Response to the COVID-19 attitude question.

The overall score of Attitudes toward COVID-19 was 32, and the average score of the respondents was 23.68 ± 3.169. Of the respondents, 28.9% had particular positive attitudes. 24.4% of the respondents were anxious about the epidemic, and 22.9% thought that the epidemic had a great impact on their lives. Regarding sex, number of household members, smoking status and chronic illnesses, dose of COVID-19 vaccine, and pre-medication, there was no statistically significant difference in the respondents’ attitudes (*p* > 0.05). With a positive attitude level of 20.5%, eastern Guangdong had the largest Attitude score gap across the regions. The respondents with the greatest Attitude score differential were those between the ages of 27 and 48, only 24.4% of whom reported having a positive attitude. A poor attitude was shown by 76.6% of respondents whose household income was less than 5,000 yuan annually. Meanwhile, respondents with the highest education in university/junior college and above, who had never drunk alcohol in the past year and had been in good health were more likely to show a positive attitude level (Figure 3; Table 2).

#### 3.2.3 Practice level of COVID-19 and its influencing factors

**Figure 4:**
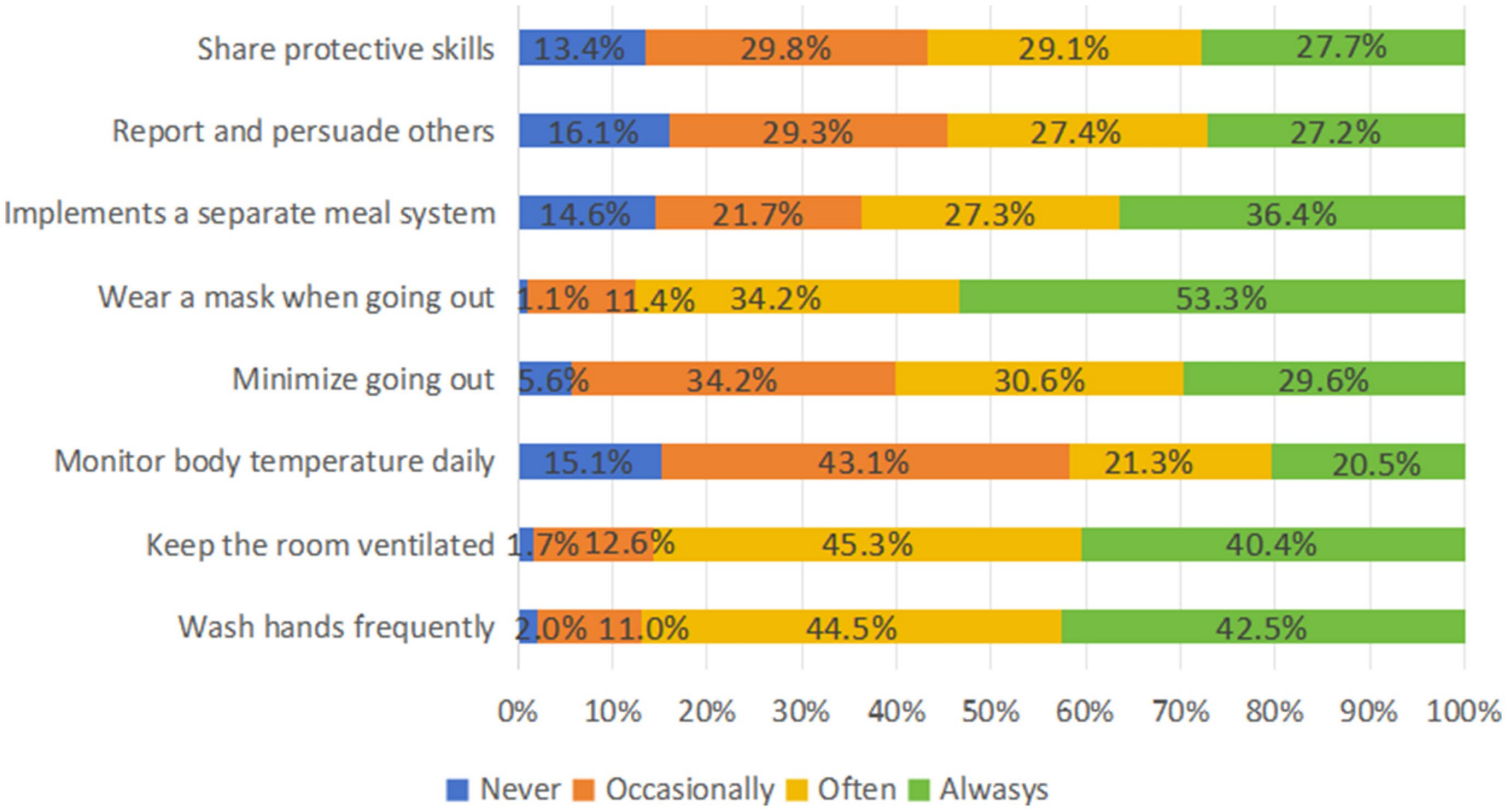
Response to the COVID-19 practice question.

The overall score of Practices was 32, and the average score of the respondents was 23.45 ± 5.030, with 33.3% reaching the level of positive practice. The results of the Chi-Square Test indicated that the participants’ Practice level was not significantly influenced by factors such as sex, number of household members, smoking status and alcohol consumption, or COVID-19 vaccination dosage (*p* > 0.05). Nearly half of respondents washed their hands frequently and wore masks outside. The respondents in western Guangdong scored their practices fairly evenly, with 296 (51.8%) having positive practices. Only a quarter of respondents exhibited protective practice, and people over the age of 49 had the highest practical differences. The largest rate of risky practice, up to 74.9%, was found among respondents whose annual *per capita* household income was less than or equal to 5,000 yuan. Respondents with no chronic diseases, good health, a high school diploma or higher, and those who had prepared their medications in advance were more likely to behave well (Figure 4; Table 2).

### 3.3 Correlation between KAP toward COVID-19 and infection

Table 3 presents the correlation between the KAP scores of individuals residing in rural regions and the infection risk during the late stage of the epidemic. This research used quartile spacing to categorize the independent variables (KAP scores) into four groups, with the reference group being comprised of the lowest quartile array. The results of the study indicated that individuals in the second, third, and highest quartiles of knowledge had a reduced risk of contracting COVID-19 compared to the reference group, even after controlling for potential confounding variables. The ORs with 95% confidence intervals for the various variables were 0.927 (0.688, 1.250), 0.722 (0.555, 0.940) and 0.691 (0.536, 0.891) respectively. A lower risk of COVID-19 was significantly associated with an increment of Knowledge score (*p* trend <0.01). In both adjusted model 2 and adjusted model 3, the risk of individuals contracting COVID-19 with Practice level at the second quartile was reduced. The odds ratio (OR) and 95% confidence intervals were 0.762 (0.596, 0.975), 0.766 (0.598, 0.980) respectively. And only in the original model, there was a clear association between the decrease in COVID-19 risk and an increase in Practice score (*p* trend <0.01). However, there was no link between Attitude score and the likelihood of SARS-CoV-2 infection in the original model or the model that was adjusted for confounding variables (*p* > 0.05).

**Table 3:** Association between infection and KAP.

Variable	Non-infected group	Infected group	Effect		
			Unadjusted OR (95% CI)^a^	Adjusted OR (95% CI)^b^	Adjusted OR (95% CI)^c^
Knowledge score					
Q1 (4 [<5])	115	408	1.00	1.00	1.00
Q2 (5 [5~])	120	409	0.961 (0.719, 1.284)	0.943 (0.701, 1.270)	0.927 (0.688, 1.250)
Q3 (6 [6~])	243	653	0.757 (0.588, 0.976)	0.739 (0.569, 0.961)	0.722 (0.555, 0.940)
Q4 (7 [7~])	327	850	0.733 (0.575, 0.934)	0.705 (0.548, 0.907)	0.691 (0.536, 0.891)
*p* trend			0.003	0.002	0.001
Attitude score					
Q1 (20 [<22])	186	532	1.00	1.00	1.00
Q2 (23 [22~])	199	586	1.030 (0.817, 1.298)	1.022 (0.804, 1.300)	1.011 (0.794, 1.287)
Q3 (24 [24~])	176	519	1.031 (0.812, 1.309)	1.050 (0.818, 1.347)	1.045 (0.814, 1.342)
Q4 (27 [26~])	244	683	0.979 (0.784, 1.222)	0.995 (0.788, 1.257)	0.990 (0.782, 1.252)
*p* trend			0.814	0.953	0.926
Practice score					
Q1 (17 [<20])	175	480	1.00	1.00	1.00
Q2 (21 [20~])	222	505	0.829 (0.656, 1.048)	0.762 (0.596, 0.975)	0.766 (0.598, 0.980)
Q3 (24 [23~])	207	660	1.162 (0.921, 1.468)	1.014 (0.793, 1.296)	1.023 (0.799, 1.309)
Q4 (30 [27~])	201	675	1.224 (0.969, 1.547)	1.117 (0.868, 1.438)	1.137 (0.882, 1.466)
*p* trend			0.010	0.084	0.060

a Model 1: unadjusted model.

b Model 2: Based on Model 1, further adjust the social demographic information (region, sex, age, annual per capita household incomes, smoking status, alcohol consumption, family population, chronic diseases, health status, highest education, etc.).

c Model 3: Further adjust the vaccination situation on the basis of Model 2.

In stratified analyses, there was a significant correlation between Knowledge scores and infection risks among Pearl River Delta respondents (*p* trend <0.01). The third quartile group and the highest quartile group had a lower observed incidence probability of SARS-CoV-2 infection compared to the reference group, with respective ORs with 95% confidence intervals of 0.624 (0.427, 0.912) and 0.525 (0.365, 0.775). Additionally, the SARS-CoV-2 infection rate is lower among individuals with Practice scores at the second quartile. In western Guangdong, compared to the reference group, residents with Knowledge scores at the third quartile have a lower SARS-CoV-2 infection rate, while residents with Practice scores at the third and highest quartiles have a higher SARS-CoV-2 infection rate. In northern Guangdong, residents with Attitude scores at the highest quartile have a higher SARS-CoV-2 infection rate. Furthermore, there is a significant association between an increase in COVID-19 risk and an increase in Attitude scores (*p* trend <0.05) (Table 4).

**Table 4:** Association between infection and KAP by region of Model 3.

Variable	Quartiles of KAP^a^				*p* trend
	Q1 (P_0_ to P_25_)	Q2 (P_25_ to P_50_)	Q3 (P_50_ ro P_75_)	Q4 (P_75_ to P_100_)	
Pearl River Delta					
Knowledge	1.00	0.865 (0.550, 1.361)	0.624 (0.427, 0.912)	0.525 (0.365, 0.757)	<0.001
Attitude	1.00	0.897 (0.635, 1.267)	0.979 (0.694, 1.381)	0.850 (0.609, 1.188)	0.375
Practice	1.00	0.697 (0.497, 0.975)	0.927 (0.658, 1.306)	1.211 (0.829, 1.769)	0.077
Eastern Guangdong					
Knowledge	1.00	0.965 (0.481, 1.936)	0.985 (0.509, 1.906)	1.108 (0.564, 2.177)	0.701
Attitude	1.00	1.182 (0.687, 2.033)	0.658 (0.353, 1.226)	0.870 (0.478, 1.584)	0.512
Practice	1.00	0.784 (0.422, 1.458)	0.798 (0.448, 1.421)	0.704 (0.380, 1.302)	0.296
Western Guangdong					
Knowledge	1.00	0.826 (0.402, 1.698)	0.502 (0.253, 0.997)	0.886 (0.415, 1.889)	0.493
Attitude	1.00	1.205 (0.580, 2.504)	1.349 (0.639, 2.848)	0.739 (0.381, 1.435)	0.211
Practice	1.00	1.984 (0.869, 4.532)	3.856 (1.726, 8.615)	2.637 (1.232, 5.646)	0.061
Northern Guangdong					
Knowledge	1.00	1.181 (0.473, 2.947)	1.657 (0.720, 3.817)	1.355 (0.665, 2.760)	0.427
Attitude	1.00	0.922 (0.460, 1.851)	1.576 (0.742, 3.349)	2.225 (1.109, 4.465)	0.016
Practice	1.00	1.524 (0.704, 3.298)	1.076 (0.512, 2.258)	1.689 (0.834, 3.424)	0.203

a Data grouped into four quartiles: Q1 (lowest 25%), Q2 (25–50%), Q3 (50–75%), and Q4 (highest 25%).

## 4 Discussion

The epidemic situation in COVID-19 has brought severe challenges to all communities and groups around the world (31, 32). As an integral component of society, the status of rural residents’ prevention and control awareness and practice is crucial to the management of the epidemic situation and risk reduction (33). Through questionnaire survey, this study investigated the Knowledge, Attitude and Practice of rural residents in China about COVID-19 epidemic in the later period of infectious diseases. The results reveal that even in the late epidemic period, the KAP score of rural residents on infectious diseases still needs to be improved, and the education and training in rural areas need to be further improved in order to better prevent and control the recurrence of infectious diseases. This contradicts the findings of these earlier studies (34, 35), which may be brought on by variations in survey duration and regional factors. According to this study, while rural residents showed a relatively good understanding of the transmission routes of infectious diseases, there was a noticeable deficiency in their knowledge regarding the sources of infection and susceptible populations. More than half of the respondents provided inaccurate answers to questions concerning the medical observation period and areas where pathogens could potentially survive for extended periods. This discrepancy may be attributed to various factors, including the educational level of rural inhabitants, their access to information, and the lag in epidemic awareness in remote areas (36, 37). Given the limited understanding of rural residents about the COVID-19 epidemic, there’s an urgent need to strengthen educational and outreach programs to minimize the risk of a disease resurgence (38).

The study highlights the complexity of KAP related to infection risks among rural residents during the later stages of the pandemic. Contrary to previous research, which suggests that higher levels of knowledge correlate with more positive attitudes toward preventative measures and a greater likelihood to implement them (39), findings from this research found that despite recognizing the significant impact of the pandemic on their lives, many rural residents harbor concerns and exhibit anxiety about the situation. This highlights the critical need to address the mental health of rural populations and the multifaceted challenges they encounter during the pandemic. The uncertainties brought about by the pandemic, coupled with concerns over health, employment, economic stability, and social pressures, contribute to increased anxiety levels (40–43). In response to the COVID-19 crisis, a variety of protective measures have been adopted within rural communities, including the use of masks, frequent handwashing, maintaining adequate indoor ventilation, and restricting outdoor activities. Despite these efforts, there remains a critical need for enhanced public health messaging, education, and improved health literacy to encourage a stronger positive attitude and effective preventive actions among rural residents (44). Such initiatives should encompass the provision of precise information and guidance, along with psychological support and coping mechanisms to alleviate the psychological toll of the pandemic, thereby contributing to the advancement of comprehensive public health security.

Furthermore, the research indicates that residents with varying features exhibit variations in their Knowledge, Attitudes, and Practices. The potential association of geographic region, age, yearly household income, and health status with the KAP level pertaining to the prevention and control of COVID-19 among rural populations in Guangdong Province is worth exploring. There are differences in KAP scores in different geographic region. Specifically, residents in northern Guangdong Province take the leading position in knowledge literacy and attitude literacy, while residents in western Guangdong Province are most outstanding in practices literacy. This phenomenon may be attributed to the remarkable effectiveness of these two regions in health education information dissemination and investment in educational resources. Due to the characteristic of a scattered population, government and health departments can implement household-by-household publicity and education more targeted, to ensure that more residents can deeply understand COVID-19-related knowledge and prevention and control measures. This research has demonstrated that individuals belonging to several age cohorts exhibit variations in their KAP toward the disease. It is possible that younger individuals tend to display heightened attentiveness toward the most recent information about the COVID-19 pandemic and subsequently adopt appropriate prevention and control strategies, while the aged population may experience cognitive limitations and physical constraints that impede their ability to engage in particular activities (45). Therefore, in publicity and education, differentiated approaches should be used for rural dwellers of all ages in order to enhance their understanding, perspective, and application of preventive and control. Consistent with other research conclusions, residents with different family economic conditions may have differences in information acquisition and protective measures (46). Families with higher incomes might have greater resources to purchase security equipment, obtain pertinent training, and take an active role in efforts to avoid and manage epidemics. Therefore, it is necessary to provide economic support and corresponding policies to ensure that all families can obtain the necessary prevention and control resources and information. Furthermore, individuals with chronic diseases or poor health may have specific challenges that hinder them from actively participating in preventive and control measures (47). These individuals require specialized care and assistance, such as individualized health education, advice on epidemic prevention, and services to encourage improved engagement in epidemic prevention and control. In order to maximize the level of KAP of rural residents with varying characteristics, it is necessary to combine the characteristics and needs of rural residents in the education and publicity work, and to develop information dissemination strategies and education programs accordingly.

A study indicated that urban residents, with greater access to information and healthcare resources, tend to have higher levels of knowledge about COVID-19 and are more likely to adopt recommended preventive behaviors (48). However, despite having less knowledge, rural residents exhibit a higher rate of correct behaviors and a positive attitude toward COVID-19 prevention measures (49). This indicates that once informed, rural residents may be more likely to adhere strongly to health directives. Furthermore, a study from South Korea has shown that knowledge directly influences attitudes and behaviors related to COVID-19, with efficacy belief acting as a significant mediating factor (50). Urban residents generally show higher KAP scores because of easier access to information and resources, while rural population may need more targeted and localized public health strategies to solve their specific needs and challenges during the epidemic. These findings underscore the importance of conducting targeted health education activities in the later stages of an infectious disease outbreak to prevent re-infection. Public health strategies must take into account the unique characteristics and needs of both urban and rural communities to effectively manage the threat of epidemics.

The investigation into the correlation between SARS-CoV-2 infection rates among rural residents and their KAP during the later stages of a pandemic is crucial for shaping effective public health strategies and intervention measures. The findings of this study demonstrate a clear link between rural residents’ comprehension of COVID-19 and their risk of infection. People with a higher knowledge level may have a lower risk of infectious diseases. People with a higher level of knowledge usually have more scientific knowledge and health awareness. They may practice better personal hygiene, adhere to health standards, and take precautions to limit the risk of infection. However, it should be noted that knowing enough about COVID-19 may not be enough to motivate people to change their practice. The results showed that after adjusting for various confounding factors, there is no direct correlation between rural residents’ attitudes scores on SARS-CoV-2 infection risk. One possible explanation is that attitude is not the key factors that directly affect the risk of infection (51, 52). Although individuals may have a good attitude and take preventive measures, other factors, such as environmental factors and social factors, may have a greater impact on the risk of infection. In addition, this study found that residents with good practice are more likely to be accompanied by lower infection risk. But after stratified analysis by region, practice turns into an irrelevant factor or even a possible risk factor. There may be other factors or mediating mechanisms that account for the lack of a clear correlation between attitude and practice scores and infection risk, such as social support, information access channels, or individual behavior motivation (53). This study found that in northern Guangdong, the trend test indicated a statistically significant trend in the infection risk as the Attitude score changed. However, residents in the highest quartile group had a relatively higher infection risk, which might suggest that overconfidence in personal protection could lead to a relaxation of vigilance in practice, thereby increasing the exposure risk. In western Guangdong, a higher preventive practice score was associated with a higher infection rate, implying that the imperfect execution of protective behaviors or exposure to high-risk social and environmental contexts might undermine the effectiveness of preventive measures. Further research is needed to explore other potential factors in order to deeply understand the relationship between attitude and practice and infection risk.

This study enhances the understanding of the KAP among rural Guangdong residents during the COVID-19 pandemic, highlighting the challenges rural areas face, and investigating the correlation between KAP and the risk of infection. Utilizing a standardized questionnaire and a significant sample size, the study guarantees the reliability of the data collected. These findings further the comprehension of epidemic patterns and are instrumental in formulating more effective public health interventions to tackle health disparities within the rural population.

However, it is important to note that this cross-sectional study captured KAP at a single point in time, which limits the ability to account for the evolving nature of the pandemic and potential shifts in KAP throughout the course of the outbreak. Moreover, the reliance on self-reported data introduces the possibility of response bias or social desirability bias, which may compromise the accuracy of the measured attitudes and practices. The study’s focus on Guangdong Province may also limit the generalizability of the findings to other rural regions in China or elsewhere. To better understand the dynamics of KAP over time and its effects on infection rates, future research would benefit from longitudinal study designs. Additionally, exploring qualitative methods could provide deeper insights into the barriers and facilitators affecting KAP in rural communities. Consideration of a broader geographical scope is also recommended to enhance the generalizability of the findings across different rural settings.

## 5 Conclusion

The study provides a comprehensive analysis of the factors that influence rural residents’ KAP concerning infection risk during the late stage of an epidemic, using COVID-19 as a case study. The findings underscore the complex interplay of socio-demographic characteristics, information access, health system factors, and individual perceptions in shaping the KAP among rural populations. The results highlight the importance of targeted educational campaigns and improved healthcare infrastructure to enhance knowledge, positively influence attitudes, and promote effective preventive practices. To address the potential barriers to improving KAP, especially in rural areas with limited resources, cultural suitability, and socio-economic conditions should be fully considered. Through health education integrating local cultural elements, the role of community workers in information transmission, and targeted economic support strategies, the popularization of health knowledge and the promotion of behavioral changes can be achieved. As rural areas often have limited resources and face unique challenges, these insights are crucial for policymakers aiming to mitigate the impact of current and future infectious disease outbreaks.

Additionally, future research could explore the role of GIS and AI services in mapping and understanding the spread of infectious diseases such as COVID-19 in rural areas. As previous studies have shown, GIS and AI have demonstrated great potential in data analysis and visualization (54–59). In the context of rural areas dealing with infectious diseases, these technologies may help map infection patterns, predict outbreaks, and allocate resources more effectively. This will contribute to formulating targeted public health policies to better protect rural communities.

## Data Availability

The raw data supporting the conclusions of this article will be made available by the authors without undue reservation.
