# Supplementary material for: Rural residents’ Knowledge, Attitude, and Practice in relation to infection risk during the late stage of an epidemic: a cross-sectional study of COVID-19
Source: Front Public Health. 2024 Dec 4;12:1450744. doi: 10.3389/fpubh.2024.1450744 (PMC11652518; doi:10.3389/fpubh.2024.1450744)
Supplement: Supplementary file 1 [file Table_3.DOCX]

**QUESTIONNAIRE (ENGLISH VERSION)**

**Survey of knowledge, attitude, practice towards COVID-19 among rural residents in Guangdong, China**

PARTICIPANT ID:

**SECTION 1: GENERAL INFORMATION ABOUT THE STUDY PARTICIPANTS**

1. Your location:
2. Pearl River Delta
3. Eastern Guangdong
4. Western Guangdong
5. Northern Guangdong
6. Your gender:
7. Male
8. Female
9. Your age in years:
10. Number of permanent residents in your household:
11. In the past year, the annual per capita income of your household was about:
12. ≤5000 yuan
13. 5001-10000 yuan
14. 10001-15000 yuan
15. 15001-20000 yuan
16. >20000 yuan
17. Have you smoked in the last three months:
18. Never smoked
19. Currently smoked
20. Quit smoking
21. Have you been drinking alcohol in the last three months:
22. Never drank alcohol
23. Currently drink alcohol
24. Quit drinking alcohol
25. Are you suffering from chronic diseases now?
26. No
27. Yes
28. Your health condition in the past year:
29. Excellent
30. Good
31. Fair
32. Poor
33. Very poor
34. Your highest educational level:
35. Elementary school or below
36. Middle school
37. High school
38. University or above
39. Have you been vaccinated against COVID-19?
40. Yes, one dose
41. Yes, two doses
42. Yes, three doses
43. Yes, four doses
44. Not vaccinated
45. Have you been infected with (had) the new coronavirus
46. Yes
47. No
48. Do you prepare medicine in advance to prevent infection?
49. Yes
50. No

**SECTION 2: QUESTIONS THAT AIMED TO ASSESS STUDY PARTICIPANT’S KNOWLEDGE ABOUT COVID-19**

1. Which of the following is the main source of infection in COVID-19?
2. Asymptomatic infections of COVID-19
3. Influenza patients
4. Healthcare workers
5. Household pets
6. Which of the following is the main mode of transmission in COVID-19?
7. Blood transmission
8. Vertical transmission
9. Contact and droplet transmission
10. Sexual transmission
11. Which of the following groups of people are susceptible to COVID-19?
12. The elderly
13. Pregnant women and children
14. Young adults
15. All groups of people
16. Symptoms that may be seen in patients with COVID-19 include：
17. Fever/dry cough
18. Nasal congestion/runny nose
19. Fatigue/pharyngeal discomfort
20. All of the above
21. When a close contacts of COVID-19 virus is isolated at home, the medical observation period is
22. 3-5 days
23. 7 days
24. 14 days
25. 18 days
26. Which of the following preventive measures is effective?
27. gargling with salt water
28. gargling with white vinegar
29. washing hands frequently and reducing exposure time
30. smoking more often
31. Which of the following operations is wrong when wearing a medical surgical mask?
32. The light-colored side should be the inner side, close to the mouth and nose, with the metal bar (nose clip) facing upwards.
33. The folded side should be completely unfolded to cover the mouth and lower jaw of the nose, and the nose clip should be pressed tightly.
34. When the inner side is wet, it can be reversed for further use.
35. Wash your hands before and after wearing the mask, and avoid touching the inside of the mask during the wearing process.
36. Which of the following is the "hardest hit" for the long-term survival of pathogens in COVID-19?
37. Woolen fabrics
38. Tap water
39. In the air
40. Smooth, non-porous hard surfaces, such as door handles, elevator buttons

**SECTION 3: QUESTIONS THAT AIMED TO ASSESS STUDY PARTICIPANT’S ATTITUDE ABOUT COVID-19**

1. You are actively concerned about the change of epidemic situation.

A. Strongly agree B. Agree C. Disagree D. Totally disagree

1. You are anxious because of the epidemic.

A. Strongly agree B. Agree C. Disagree D. Totally disagree

1. You admire the frontline anti-epidemic medical staff.

A. Strongly agree B. Agree C. Disagree D. Totally disagree

1. You are willing to take part in voluntary activities to fight the epidemic.

A. Strongly agree B. Agree C. Disagree D. Totally disagree

1. You have confidence in this epidemic control.

A. Strongly agree B. Agree C. Disagree D. Totally disagree

1. You think the epidemic has a great impact on your life.

A. Strongly agree B. Agree C. Disagree D. Totally disagree

1. You agree that the state has adjusted the level of epidemic prevention and control in COVID-19.

A. Strongly agree B. Agree C. Disagree D. Totally disagree

1. You are willing to mobilize friends to respond to national epidemic prevention measures.

A. Strongly agree B. Agree C. Disagree D. Totally disagree

**SECTION 4: QUESTIONS THAT AIMED TO ASSESS STUDY PARTICIPANT’S PRACTICE ABOUT COVID-19**

1. Do you keep washing your hands frequently?

A. Never B. Occasionally C. Often D. Always

2. Do you keep the room ventilated?

A. Never B. Occasionally C. Often D. Always

3. Do you monitor your temperature every day?

A. Never B. Occasionally C. Often D. Always

4. Do you go out as little as possible?

A. Never B. Occasionally C. Often D. Always

5. Do you keep wearing a medical surgical mask when you have to go out?

A. Never B. Occasionally C. Often D. Always

6. When a family member is infected with COVID-19, do you practice separate meals at home?

A. Never B. Occasionally C. Often D. Always

7. When you find suspicious patients around you, will you report to the management and advise them to see a doctor as soon as possible?

A. Never B. Occasionally C. Often D. Always

8. Have you introduced your protective skills to your family/neighbors/friends?

A. Never B. Occasionally C. Often D. Always
